# Genetic and clinical characterization of 73 Pigmentary Mosaicism patients: revealing the genetic basis of clinical manifestations

**DOI:** 10.1186/s13023-019-1208-0

**Published:** 2019-11-15

**Authors:** C. Salas-Labadía, S. Gómez-Carmona, R. Cruz-Alcívar, D. Martínez-Anaya, V. Del Castillo-Ruiz, C. Durán-McKinster, V. Ulloa-Avilés, E. Yokoyama-Rebollar, A. Ruiz-Herrera, P. Navarrete-Meneses, E. Lieberman-Hernández, A. González-Del Angel, D. Cervantes-Barragán, C. Villarroel-Cortés, A. Reyes-León, D. Suárez-Pérez, A. Pedraza-Meléndez, A. González-Orsuna, P. Pérez-Vera

**Affiliations:** 1 Laboratorio de Genética y Cáncer, Departamento de Genética Humana, Instituto Nacional de Pediatría, 04530 Ciudad de México, Mexico; 2 Departamento de Genética Médica, Centro de Rehabilitación e Inclusión Infantil Teletón, Tuxtla Gutiérrez, Chiapas Mexico; 3 Departamento de Genética Humana, Instituto Nacional de Pediatría, Ciudad de México, Mexico; 4 Laboratorio de Citogenética, Genos Médica, Centro Especializado en Genética, Ciudad de México, Mexico; 5 Facultad de Ciencias, Universidad Nacional Autónoma de México, Ciudad de México, Mexico; 6 Departamento de Dermatología, Instituto Nacional de Pediatría, Ciudad de México, Mexico; 7 Hospital de Especialidades Pediátrico de León, León, Guanajuato, Mexico; 8 Laboratorio de Biología Molecular, Departamento de Genética Humana, Instituto Nacional de Pediatría, Ciudad de México, Mexico; 9 Hospital Central Sur de Alta Especialidad, PEMEX, Ciudad de México, Mexico; 10 Posgrado en Ciencias Biológicas, Universidad Nacional Autónoma de México, Ciudad de México, Mexico

**Keywords:** Pigmentary mosaicism, Cytogenetic and molecular characterization, Genotype-phenotype correlation

## Abstract

**Background:**

Pigmentary mosaicism constitutes a heterogeneous group of skin pigmentation alterations associated with multisystem involvement. The aim of this study was to establish a complete cytogenetic and molecular characterization of PM patients, emphasizing on searching for possible low chromosomal mosaicism and on establishing an accurate genotype-phenotype correlation.

**Results:**

A total of 73 patients were included (3 months to 18 years of age), 52% male and 48% female. Observed in 69 (95%) patients, the most frequent pattern of pigmentation was fine and whorled BL, which was associated with disseminated skin extent in 41 (59%) patients. Central nervous system (84%) alterations were the most frequent observed in the group of patients, followed by the musculoskeletal (53%) and ophthalmologic (27%) alterations. Considering the pattern of pigmentation, no significant differences in association with skin extent or extracutaneous manifestations were detected. Following a strict cytogenetic analysis strategy, screening metaphases from three different tissues (peripheral blood, hyperpigmented and hypopigmented skin) we found that 23/73 patients had chromosomal abnormalities classified as follows: 1) Mosaic with 2 or more different cell lines with structural alterations *n* = 19; 2) Polyploidy (mosaic) *n* = 1 and 3) Alterations in all cells in three different tissues *n* = 3. SNP array, array CGH and FISH were useful for the complete characterization of the chromosomal aberrations, for the detection of microdeletions in patients with normal karyotype but with strong clinical suspicious of chromosomal alteration, and for a better establishment of genotype-phenotype correlation. In 2 patients we found genes associated with some of the extracutaneous manifestations (*SHH, MNX1, PPP2R2C*).

**Conclusions:**

This group of 73 patients finely described is the largest series of patients with pigmentary mosaicism reported worldwide. As we showed in this study, the followed analysis strategy allowed the detection of cytogenetic and molecular abnormalities, and made possible the establishment of genotype-phenotype associations in some patients. An important limitation of our study was the analysis of fibroblasts cultures instead of melanocytes and keratinocytes. In some cases the direct molecular DNA analysis of skin biopsy could be another choice.

## Background

**Fig. 1:**
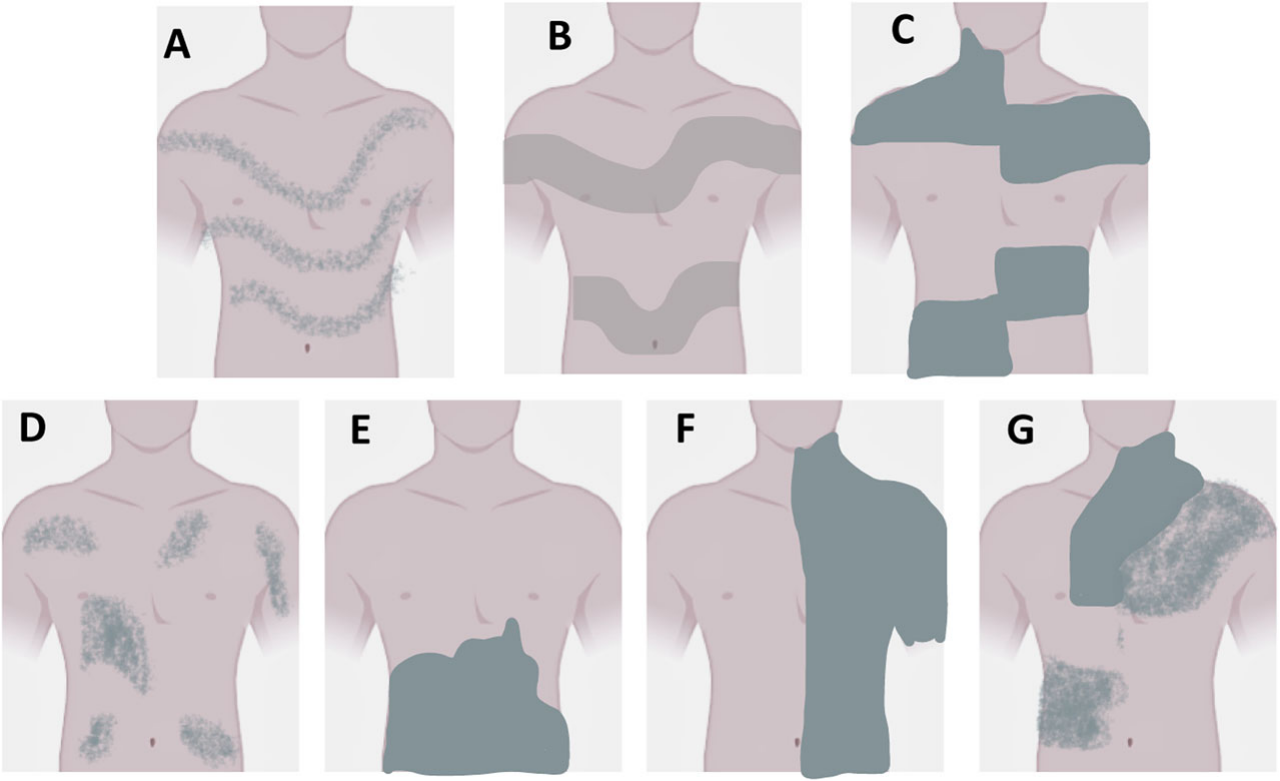
Pigmentary Mosaicism Arquetypes. **a**) Narrow bands Blaschko lines; **b**) Broad bands Blaschko lines; **c**) Checkerboard pattern; **d**) Phylloid pattern; **e**) Patchy pattern without midline separation; **f**) Lateralization pattern and **g**) Sash-like pattern

Pigmentary mosaicism (PM) of the hypomelanosis of Ito type (IH) (OMIM#300337) or of the linear and whorled nevoid hypermelanosis type (LWNH) (OMIM#614323) constitutes a heterogeneous group of skin pigmentation disorders characterized by the presence of hypopigmented and hyperpigmented macules, which follow patterns of cutaneous mosaicism as evidence of migration routes of melanocytes and keratinocytes during embryogenesis, known as Blaschko lines (BL). The cutaneous mosaicism can occur in 6 different archetypical patterns including the fine and whorled and the broad BL pattern, and depending on the part of the body where it occurs (cell type), they are distributed differently (Fig. 1) [1–3]. It is important to note the existence of less-well defined patterns as the Pallister-Killian pattern, the mesotropic facial pattern and a third described as cutis tricolor like [4, 5].

In IH or LWNH PM type patients, the different skin pigmentation patterns may appear as a unique manifestation or in association with extracutaneous manifestations, mainly of the central nervous system (CNS), so it has been characterized as a neurocutaneous disorder [6–9]. Multisystem involvement has been observed in 30–90% of PM patients, and the most common findings are intellectual disability (ID) and seizures in 40–60% of PM patients. Ocular abnormalities, musculoskeletal deformities (M-S) and dysmorphic facial features (DFF) are other frequent findings [8, 10–14].

The presence of cytogenetic alterations as the pathogenic basis of PM has been key to explain the wide variability of clinical manifestations in these patients. Several chromosomal aberrations (polyploidy, aneuploidies, marker chromosomes, rings, deletions and translocations) have been observed in 30–60% of PM cases when both leukocytes and cultured fibroblasts are evaluated in the same patient, and in more than 80% of the cases chromosomal abnormalities are present in mosaic state [3, 12, 14–17]. It is assumed that the presence of differential skin pigmentation could be related with two distinct genotypes in each type of skin [2]. Different types of genetic pathogenic variants have also been reported, such as microdeletions and mutations in genes associated with skin pigmentation and the presence of other associated abnormalities [12, 16–19]. The high proportion of genetic mosaicism observed in PM patients, together with the great variability at clinical and genetic levels, highlights the importance to establish a strategy that increases the probability of finding genetic abnormalities even in mosaic state, for a better characterization of this entity. The aim of this study is to achieve a complete cytogenetic and molecular characterization on the largest group of patients with PM, emphasizing on searching for possible low chromosomal mosaicism and on establishing an accurate genotype-phenotype correlation.

## Subjects and methods

We included 73 pediatric patients with PM with or without other systemic anomalies referred from four different hospitals in Mexico from 2006 to 2018, and diagnosed by Genetics and Dermatology departments. Information on sex, age, dermatological findings, such as a) type of pigmentation: hypopigmentation / Light skin (LS), hyperpigmentation / Dark skin (DS) or both; b) pigmentation pattern: fine and whorled or broad BL and skin extent of dermatosis, as well as precise data associated with extracutaneous manifestations, were obtained for each patient. It worthwhile to highlight that all of the extracutaneous manifestations were analyzed by medical specialists depending on the system affected (e.g. neurologist for CNS alterations). This study was approved by Research Ethics committee with National Commission of Bioethics registration number “CONBIOETICA-09-CEI-025-20,161,215” of Instituto Nacional de Pediatría. Signed informed consent forms were obtained according to the recommendations of the Helsinki Declaration.

As a standardized protocol, all PM patients with pigmentary abnormalities associated or not with extracutaneous manifestations were analyzed as follows: 1) Cytogenetic analysis in peripheral blood lymphocytes (PB) and in fibroblasts cultures obtained from LS and DS following standard methods. G-banded metaphases were interpreted according to the International System for Human Cytogenetic Nomenclature 2016 [20]. A total of 50 GTG-metaphases were analyzed per tissue following a strict criteria analysis. 2) Molecular analysis with SNP array or array CGH (aCGH) and/or fluorescence in situ hybridization (FISH) was performed only in patients in whom a better characterization of chromosomal abnormalities (CA) was needed. 3) This analysis also included some patients with normal karyotype, but strong clinical suspicion (ID, DFF, PM and alterations in other involved systems) of having a chromosomal alteration below the detection level of classical cytogenetics. DNA for aCGH was isolated from PB, LS and DS using the specific Qiagen Kit according to the manufacturer’s instructions. aCGH 60, 100, 180, 400 k and 750 k was carried out using Agilent Technologies (Santa Clara, CA), which contains between 60,000, and 750,000 probes, distributed between coding and non-coding human genome sequences (hg18, hg19 UCSC). SNP array genotyping was carried out using the Illumina BeadStation (Human Hap550 BeadChip (V3)). Loss of genomic regions was determined by decreases in the log R ratio. Mosaic proportions were estimated by B allele frequencies (BAF) [21, 22].

At the clinical level and for later comparison purposes, the body regions involved in the pigmentation changes were divided in four segments: head, trunk and upper and lower limbs. Localized dermatosis was considered when only one segment was involved, and disseminated dermatosis when 2–3 segments were involved.

Pigmentation pattern, pigmentation type frequencies, and its association with skin extent dermatosis and extracutaneous manifestations were evaluated by Fisher exact test (SPSS 20.0). Presence or absence of chromosomal abnormalities, were associated with the distribution of pigmentation pattern and extracutaneous manifestations. *P*-values less than 0.05 were considered significant.

## Results

### Study population

**Fig. 2:**
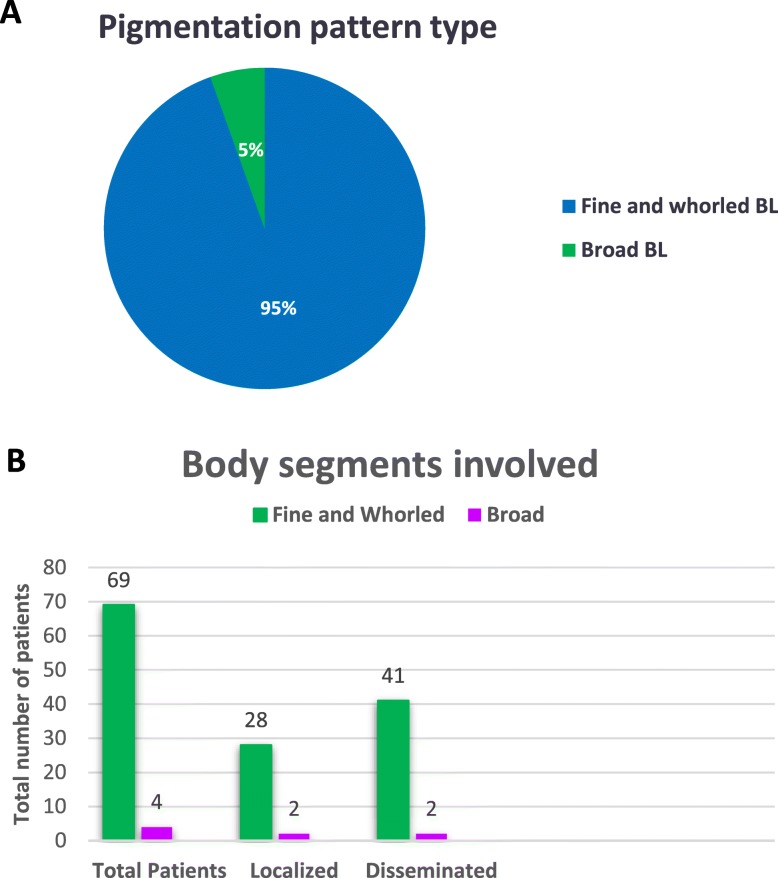
Pigmentation pattern. **a** Type of pattern pigmentation: Fine and whorled BL (*n* = 69), and Broad BL (*n* = 4). **b** Body segments involved in association with pattern pigmentation: localized (1 segment, Fine and whorled BL *n* = 28; Broad BL *n* = 2), disseminated (2–3 segments, Fine and whorled BL *n* = 41; Broad BL *n* = 2)

**Fig. 3:**
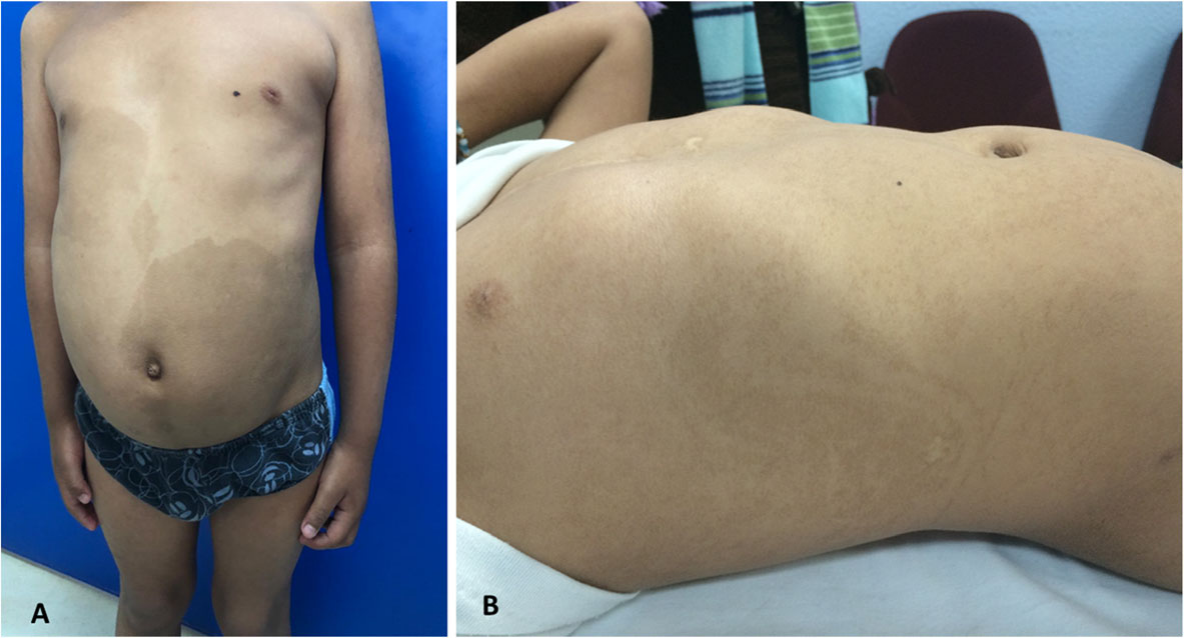
**a** Patient with hyperpigmented Broad BL pattern; **b** Patient with hypopigmented fine and whorled BL pattern in trunk

A total of 73 patients with PM were included, 52% were male and 48% were female. Age at the time of diagnosis ranged from 3 months to 18 years. Hypopigmentation was observed in 14 (19%) patients, hyperpigmentation in 31 (43%) and combined patterns in 28 (38%). The more frequent patterns of pigmentation were fine and whorled BL observed in 69 (95%) patients, followed by broad BL pattern in 4 (5%) patients (Fig. 2). The most frequently observed dermatosis was the disseminated, which was detected in 43 patients (59%), followed by the localized, observed in 30 patients (41%). The fine and whorled BL pattern associated with disseminated skin extent was the most frequently detected dermatosis, identified in 41 patients (59%). The other 28 (41%) patients presented a localized pattern (Fig. 2).Two patients showed a localized and two a disseminated pattern associated with the broad BL pattern. No significant difference was detected between different BL pattern and skin extent (*p = 0.5)*. Representative photographs of patient with hyperpigmented Broad BL pattern and patient with hypopigmented fine and whorled BL pattern in trunk are included (Fig. 3).

### Extracutaneous manifestations

**Fig. 4:**
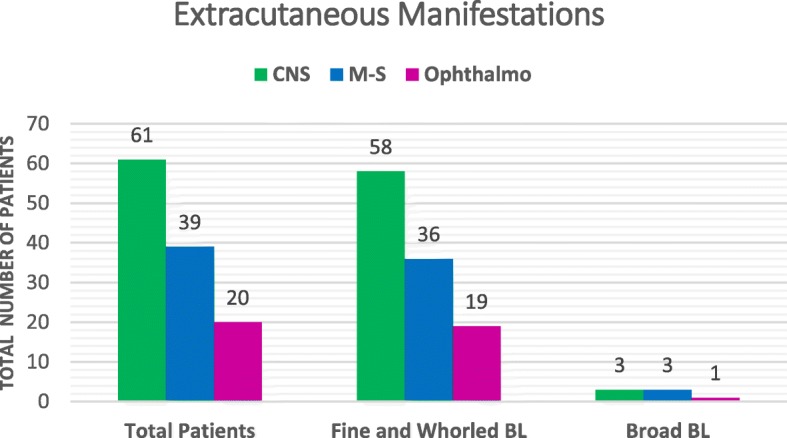
Extracutaneous Manifestations. Total of patients with abnormalities in different systems, and distribution associated with pattern pigmentation type: Fine and whorled BL and Broad BL. CNS Central Nervous System; M-S Musculoskeletal

As reported in the literature, the CNS was the most frequently involved system in the group of patients. A total of 61/73 (84%) patients had CNS alterations including: ID, seizures, hypotonia, microcephaly, and/or abnormal EEG (epileptiform discharges: seizure activity), and MRI (focal cortical dysplasia associated alterations: heterotopia, lissencephaly and polymicrogyria). Regarding the pattern of pigmentation, 58/61 (95%) patients with fine and whorled BL and 3/61 (5%) with broad BL had CNS manifestations (*p = 0.48*) (Fig. 4). The second system most frequently involved in 39/73 patients (53%) was the musculoskeletal system (M-S), and the hemihypertrophy was the most representative manifestation, followed by scoliosis, digital alterations and hip dysplasia. Among these 39 patients with musculoskeletal alterations, 36 (92%) presented fine and whorled BL pattern and 3 (8%) showed broad BL (*p = 0.36*) (Fig. 4). The third most frequently involved system was the ocular system in 20/73 patients (27%). In this case ocular manifestations such as microphthalmia, strabismus, ptosis and cataracts were the most representative. Of the 20 patients with ocular abnormalities, 19 (95%) presented fine and 1 (5%) broad BL pattern (Fig. 4). Considering the pattern of pigmentation, no significant difference in association with ophthalmologic manifestations was detected *(p = 0.69)*. Finally, low weight, short and tall stature, endocrine manifestations such obesity and precocious puberty, DFF, hearing loss and involvement of cardiac, genitourinary, and digestive systems were also detected. Only 5/73 patients (7%) did not show any extracutaneous manifestation. Importantly, 28/68 (41%), 35/68 (52%) and 5/68 (7%) of patients with extracutaneous manifestations had abnormalities involving 1–2, 3–5, and more than 6 different systems, respectively. We observed statistically significant differences between groups, accordingly to the presence of other clinical manifestations in one or more systems affected in association with PM (*p < 0.001*).

### Cytogenetic analysis

Cytogenetic analysis on 50 metaphases for each tissue was obtained. The analysis of the three different tissues was achieved for most patients (59/73, 81%). For the remaining patients at least two different tissues were analyzed, both skins without PB in 12/73 (16%) patients, and PB and LS or DS in 2/73 (3%) patients.

**Fig. 5:**
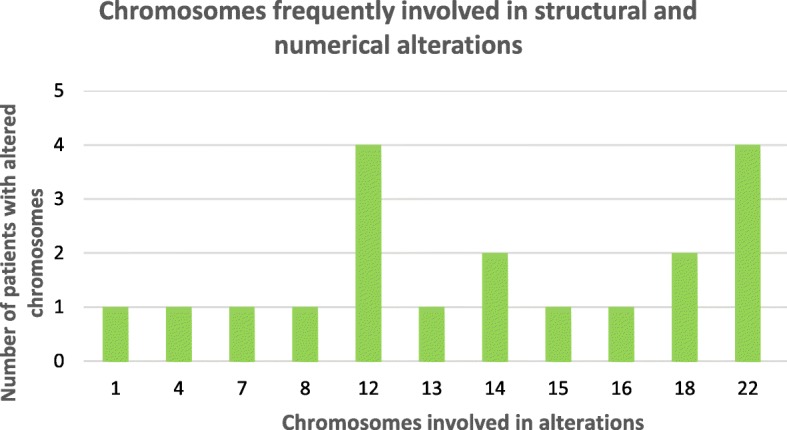
Chromosomes involved in alterations. Total of structural and numerical chromosomal abnormalities for each chromosome

The cytogenetic analysis showed abnormal karyotypes in 23/73 (31%) patients. The frequency of patients with mosaic chromosomal abnormalities in three, two or only one tissue was 45, 35 and 20%, respectively. Only 3 patients had non-mosaic chromosomal abnormalities (Table 1). Polyploidy in mosaic was observed in one patient (Table 1). The chromosomes involved in the abnormalities were 1, 4, 7, 8, 12, 13, 14, 15, 16, 18, 22, X, for both numerical and structural rearrangements. Chromosomes 12 and 22 presented numerical and structural rearrangements in 4 patients each (Fig. 5). The detailed descriptions of the abnormal karyotypes detected are listed in Additional file 1.

**Table 1:** Cytogenetic findings

TOTAL PATIENTS	73
Cytogenetic analysis	73
Normal Karyotype	50
Chromosomal alterations:	
1) Mosaic with 2 or more different cell lines with structural alterations	
a) Mosaic in 3 tissues analyzed: PB, LS and DS	
^a^mos − 7/r(7)(p22q36.3)/normal	1
mos del(18)(q21.3)/normal	1
mos r(22)(p11.2q13.2)/normal	1
^a^mos + del (14) (q11.2)/+ 14/normal	1
^a^mos + der(X)(p21.1p11.1)/45,X/normal	1
^a^mos + del(14)(q11.1)/normal	1
^a^mos + idic(15)(q13.3)/normal	1
Total	7
b) Mosaic in LS and DS / PB normal	
Skin: mos + i(12)(p10)/normal	1
DS: mos t(1;8)(p?36;q?22)/normal	1
Skin: mos + mar/normal	1
Skin: mos + 12/normal	3
^a^Skin: mos −18,+der(18)?del18/normal	1
Skin: mos + mar1/+mar2/−12, +mar2/+mar1, +mar2/normal	1
DS: mos +?22[3]/normal	1
Total	9
c) Mosaic in PB / Skin normal	
PB: mos + mar/normal	1
Total	1
d) Mosaic in skin / PB, DS or LS all cells with alteration	
mos r(22)(p11.2q13.2)/idic (22) (p11.2q13.2)/normal	1
PB: r(22)(p11.2q13.3)	
mos add(16)(p13.3)/normal	1
DS: add(16)(p13.3)	
Total	2
2) Polyploidy (Mosaic)	
mos 69,XXY/46,XY	1
Total	1
3) Alteration in all cells / 3 different tissues	
t(X;22)(q11.2;q13.3)	1
^a^del(13)(q21.3q32.1)	1
^a^del(4)(p16.1p15.3)	1
Total	3
Total of Patients with chromosomal alterations	23

*PB* Peripheral Blood, *LS* Light Skin (hypopigmented), *DS* Dark Skin (hyperpigmented)

^a^Patients with molecular analysis

### Molecular analysis

The SNP array analysis or aCGH 60, 100, 180 and 400 k as well as FISH analysis with different probes were applied in different situations. In a subgroup of patients: PM2, PM25, PM30, PM39, PM46, PM53, PM61 and PM65, these tools were useful for detecting abnormal clones, and to complete the characterization of the chromosomal aberrations found by classical cytogenetics (See additional file 1). In another subgroup of patients with normal karyotype, but altered phenotype with ID, DFF and PM, besides the involvement of alterations in other systems, the molecular analysis by SNP array and aCGH 60 and 750 k resulted normal (6/7 patients (PM20, PM32, PM38, PM45, PM54 and PM77)). Only one patient (PM35) with normal karyotype showed a pathogenic 0.6 Mb deletion (16p11.2) containing more than 20 genes. Lastly, in two other patients molecular analysis was performed. In patient PM27 with a cytogenetic abnormality in eleven cells of DS, the SNP array analysis did not show any alteration, probably because it is a balanced alteration not detected by this methodology. In the second patient (PM42) with a marker chromosome previously detected by cytogenetic analysis, the alteration could not be further defined by aCGH 60 K, suggesting that the marker chromosome was mainly heterochromatic. However, as the alteration was found in a low number of cells, it is probable that the resolution of the microarray was not enough for detecting it (See additional file 1).

### Distribution of pigmentation pattern and extracutaneous manifestations in patients with and without chromosomal abnormalities

Of the 23 patients with chromosomal abnormalities, 21 (91%) presented fine and whorled BL and 2 (9%) broad BL pattern. In the group of patients with chromosomal abnormalities, the disseminated pigmentary pattern was observed in 18/23 patients (78%), in comparison with the localized pattern, that was only observed in 5 patients, showing to be statistically significant (*p = 0.01*).

In addition, of the 23 patients with chromosomal alterations, 22 (91.6%) had CNS manifestations, 14 (58.4%) were associated with alterations of the M-S system, and 6 (25%) with ocular alterations. No significant difference was detected in the distribution of extracutaneous manifestations between patients with or without chromosomal alterations (*p = 0.20; p = 0.80; p = 0.90* respectively).

### Genotype-phenotype correlation: relevant examples

In some patients, it was possible to establish a genotype-phenotype correlation associated with cytogenetic and molecular analysis results. As an example, in patients PM2, PM25, PM30 and PM53, the cytogenetic analysis showed a specific chromosomal abnormality in each one (Table 2). By molecular analysis it was possible to accurately characterize each alteration, and determined the probable origin of the clinical manifestations (Table 2).

**Fig. 6:**
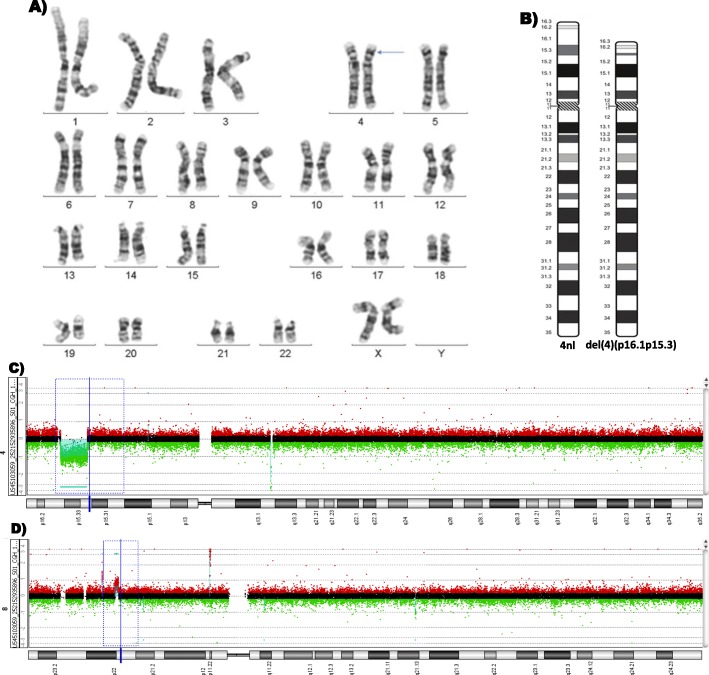
Cytogenetic and molecular analysis of patient PM53. **a** Karyotype with del(4)(p16.1p15.3) (blue arrow); **b** Ideogram of normal (4 nl) and del(4)(p16.1p15.3) chromosomes; Microarray CGH Agilent 100 K, hg19 showing **c**) Deletion of 7.5 Mb in 4p16.1-p15.32 (green arrow) and **d**) Microduplication of 0.81 Mb in 8p22-p21.3 (red arrow)

The cytogenetic analysis of patient PM2 showed the presence of mosaicism with r(7), monosomy 7 and duplicated r(7) in 3 different tissues. A normal cell line was not detected. Analysis by SNP array showed a 0.8 Mb deletion in 7p22.3 (involving eight genes) and a 7.5 Mb deletion in 7q36 (involving 29 genes including some involved in genital and central nervous system development). As shown in Table 2, most of the deleted genes were involved in CNS, urogenital and M-S systems alterations associated with some of the clinical manifestations of the patient (Table 2) [21].
2) Cytogenetic analysis identified three cell lines in different proportions in patient PM25: One with supernumerary marker chromosome, another with trisomy 14, and a normal cell line. aCGH 180 k confirmed the cytogenetic marker chromosome as del(14)(q11.2). Considering the negative results of phenotype genes association with marker chromosome and maternal UPD analysis of previous study, the phenotype of the reported patient apparently correlate with mosaic trisomy 14 (Table 2) [23].
The initial cytogenetic analysis of patient PM30 showed a mosaic karyotype including monosomy X and a supernumerary chromosome of unknown origin. FISH analysis with the α satellite X probe confirmed that the marker was a derivative X chromosome. The SNP array further confirmed the mosaic karyotype with a der(X) chromosome duplicated at Xp21.1p11.1 region (Table 2). The patient’s phenotype could be classified as a variant of Turner syndrome because of the partial functional disomy of the X chromosome. As reported in the literature, other patients with this functional disomy have ID and DFF (Table 2) [24].
The cytogenetic analysis of patient PM53 showed an interstitial del(4)(p16.1p15.3) in PB, LS and DS in all cells analyzed. aCGH100k confirmed the cytogenetic alteration as del(4)(p16.1p15.3). This deletion encompasses 7.5 Mb outside of the Wolff-Hirschhorn locus, and involves 30 genes. In this patient loss of the *PPP2R2C* gene was associated with the ID. Also, a 0.81 Mb duplication in chromosome 8 (not associated with phenotype) was detected. (Table 2 and Fig. 6) [25].

**Table 2:** Genotype-Phenotype correlation

Patient Code	PM Pattern	Extracutaneous Manifestations	Cytogenetic Analysis	Molecular Analysis	Association with clinical manifestations
^a^PM2	Broad BL with disseminated hypo and hyperpigmentation in face, dorsum and limbs	ID, ptosis, sacral defects, scoliosis, hypoplastic genitalia, small testes and low weight and length	PB: mos 46, XY, r(7) (p22q36.3) [45]/45, XY,-7 [6]/46,XY, dup(7)(p22q36.3) [2] LS: mos 45, XY, −7 [23]/46, XY, r(7) (p22q36) [17] DS: mos 45, XY, −7 [26] /46, XY, r(7) (p22q36) [29]	1. arr 7p22.3(113,336–954,145)×1,7q36.1q36.3 (151306863–158,812,247) × 1, (Human Genome Build, hg18). 2. FISH analysis with subtelomeric probe for chromosome 7: ish r(7)(p22q36.3) (VIJyRM2185-,VYJyRM2000-)	1) 0.8 Mb deletion in 7p22.3 including 8 genes associated with urogenital anomalies 2) 7.5 Mb deletion in 7q36.1: 50 RefSeq genes. Phenotype related: a) *SHH*, eye anomalies (ptosis in present case) b) *MNX1*, sacral defects (e.g. Currarino Syndrome: partial fusion S2-S5 vertebrae) (21)
^a^PM25	Fine and whorled BL with disseminated hypo and hyperpigmentation in dorsum and limbs	ID, DFF, scoliosis, hip dysplasia, and low weight	PB: mos 47,XX,+mar [45]/47,XX, + 14 [10]/46,XX [45] LS: mos 47,XX,+mar [7]/46,XX [8] DS: mos 47,XX,+mar [12]/46,XX [14]	1. arr 14q11.1q11.2(18,127,052-19,927,052)×2~3, (UCSC, h18) 2. FISH with DNA BAC probes for 14q11.2 spectrum green and 14q32.33 spectrum Final result: PB: mos 47,XX,+del(14)(q11.2)[45]/ 47,XX,+ 14[10]/46,XX[45]	Phenotype associated with mosaic trisomy 14 (23)
PM30	Fine and whorled BL with hypo and hyperpigmentation in dorsum and inferior limbs	Severe ID, seizures, hypotonia and DFF.	PB: mos 47, XX,+mar [28]/45,X [6] / 46, XX [16] LS: mos 45,X [35] / 47, XX,+mar [2]/ 46, XX [6] DS: mos 45,X [20] / 47, XX,+mar [3]/ 46, XX [13]	1. arr Xp22.33q28(60,814-155,254,881)× 1[~ 30%], Xp21.1p11.1(36,025,401-58,483,247)×3 [~ 55%] (Human Genome Build 37, hg19) 2. FISH with α-satellite X probe (AbbotMolecular/Vysis) and locus specific probe (p11.22-p11.23) (Agilent Tech, SureFISH) positive. Final result: PB: mos 47, XX, +der(X)(p21.1p11.1) [28]/45,X [6] / 46, XX [16]	Variant of Turner syndrome, severe phenotype as a result of partial functional disomy. Associated in previous study with ID and DFF (24)
PM53	Fine and whorled BL with hypo and hyperpigmentation in dorsum and inferior limbs	ID, DFF	PB: 46,XX,del(4)(p16.1p15.3) [50] LS: 46,XX,del(4)(p16.1p15.3) [50] DS: 46,XX,del(4)(p16.1p15.3) [50]	1. arr 4p16.1p15.32 (10,047,353-17,614,303)× 1, 8p22p21.3 (18,712, 712–19, 523, 339)× 3, (UCSC, hg19)	1. 0.81 Mb duplication including 3 genes in chromosome 8 without phenotype correlation 2. 7.5 Mb deletion including 30 genes. Phenotype related: a) *PPP2R2C* associated with ID (25)

*MP* Pigmentary Mosaicism, *BL* Blaschko Lines, *ID* Developmental Delay, *DFF* Dysmorphic Facial Features, *M-S* Musculoskeletal alterations, *PB* Peripheral Blood, *LS* Light Skin (hypopigmented), *DS* Dark Skin (hyperpigmented). ^a^Patients previously reported

## Discussion

To date, this group of 73 patients constitutes the largest clinical and cytogenetically finely described study of cases with PM of the IH type or LWNH type, that were selected consecutively for 13 years. The pigmentary abnormalities were the main inclusion criterion, associated or not with extracutaneous manifestations.

PM is an entity mainly characterized by skin pigmentary alterations, and in this group of patients, the hyperpigmentation in fine and whorled BL pattern was the most frequently observed (49%). In contrast, in 5 previous reports the hypopigmentation was the most frequent type of pigmentation in PM (ranging 50–100%) [1, 3, 6, 26, 27]. Only one previous report identified hyperpigmentation as the most frequently group (77%) [7]. It is important to notice that the combined pattern (hypo/hyperpigmentation) was the group with the lowest frequency of presentation in almost all previous studies. However, in our group the combined pigmentation pattern was as frequent as the hyperpigmentation subgroup.

Our study revealed a total of 68 (93%) patients with extracutaneous manifestations. CNS was the most frequently involved system, followed by M-S and ophthalmologic systems. At CNS level, ID and seizures were the most common findings, as most studies have previously shown [3]. In the M-S system the asymmetric overgrowth was the most representative manifestation, observed in 11/73 (15%) patients. It is an important finding considering that in 5 previous studies, the combination of PM with asymmetric overgrowth was reported in 6/6(100%), 8/114 (7%), 4/34 (12%), 4/19 (21%) and 12/76 (16%) patients [1, 28–31]. Thus, taking together with our results, it can be appreciated that the association of PM with asymmetric overgrowth is not uncommon. Other studies showed the ophthalmologic system as the third most frequently involved in PM, as in this group of patients [3, 8, 13, 16, 18] .

After a thorough cytogenetic analysis, the frequency of patients with chromosomal abnormalities was found to be 31%, all of them with extracutaneous manifestations. In total, nineteen patients had mosaic with 2 or more cell lines with structural alterations in different tissues. Non-mosaic chromosome alteration was found in 3 patients, and polyploidy in one patient. In a previous review study, the total frequency of patients with chromosomal abnormalities was 43%, with the presence of mosaic state abnormalities being the most representative [3]. In present study, the chromosomes more frequently involved in abnormalities were 12 and 22, for both numerical and structural alterations. Although there are chromosomes that are involved more frequently in abnormalities, the general spectrum of chromosomes that are found in altered karyotypes is very broad; this demonstrates the need to diagnose at genetic level patients with entities as PM of IH type, LWNH type or others [3]. Then, we consider that screening a large number of metaphases from three different tissues, increases the probability to know the etiology of PM. This strategy allows: 1) To detect different proportion of cells with chromosomal abnormalities; 2) To rule out if the alteration is confined only to a single tissue, and 3) To find an alteration by increasing the number of cells analyzed per every tissue.

The presence of non-mosaic chromosomal rearrangements in 3 patients and its association with pigmentary manifestations could be explained as follow: 1) t(X;22)(q11.2;q13.3). There is a subgroup of female patients with PM of IH type with the presence of constitutional X;autosome translocation. The phenotype in these cases results from the presence of mosaic functional disomy of X chromosome, as a consequence of normal X inactivation process during embryogenesis [3, 32]. 2) And in the other 2 patients with deletions in chromosomes 4 and 13, the chromosomal alteration itself does not explain the PM manifestations, however as described above (Table 1) could be related with some of the extracutaneous manifestations as CNS alterations [25].

An important limitation of our chromosomal analysis is the use of fibroblasts culture instead of using keratinocytes or melanocytes. However, Taibjee et al., 2009 studied patients with clinically suspected PM and normal blood cytogenetics. They analyzed fibroblasts and keratinocytes to identify chromosomal mosaicism, and the results seem to be insufficient to justify routine keratinocyte cytogenetic investigation [33]. Another option could be the analysis by SNP array or next generation sequencing (NGS) of genomic DNA directly extracted from hypopigmented and hyperpigmented skin biopsies; however, we must consider that with these molecular methodologies some of genetic abnormalities could be missed [34].

The disseminated fine and whorled BL dermatosis was present in 95% of PM patients, and considering patients with chromosomal alterations, the same pattern was presented in 78% of cases. More than 50% of patients with extracutaneous manifestations presented more than two different systems affected. In general, the subgroup with combined pattern of pigmentation had the highest number of patients with involvement of at least three different systems. It is important to note that it seems that the more complex the pattern of distribution (disseminated) and type of pigmentation (combined pattern), the greater the number of different affected systems in the same patient and at the same time with the presence of chromosomal alterations. It has been proposed that this is a result of the presence of a greater number of ectodermal cells with some genetic defect associated with multiple manifestations in one patient [16]. However, related with the pigmentation pattern we cannot assume the same affirmation because only 4 patients with broad BL pattern were studied.

Accordingly to the recently described interpretation of pigmentation pattern type from an embryological point of view, PM patients were regrouped in fine and whorled BL pattern and in broad BL pattern, to allow a possible association with the manifestation of extracutaneous alterations [34, 35]. However in our study, and analyzing only the regrouped patients (fine and whorled BL patients group vs. broad BL patients group), no differences were observed between groups, considering skin extent of dermatosis, extracutaneous manifestations and chromosomal abnormalities associated.

Molecular analysis has also proven to be a useful tool in: 1) Detecting abnormal cell clones and 2) Completing the characterization of the chromosomal aberrations identified by classical cytogenetics. Moreover, in one patient with normal karyotype the molecular analysis detected a microdeletion in 16p11.2, this alteration has not been associated with a clearly defined phenotype but it has been reported in patients with mild intellectual disability, autism, obesity, seizures and cardiac defects. It is important to consider that the presence of other genetic changes like point mutations in genes associated with the pigmentary alterations, and some of extracutaneous manifestations, could be related with the clinical characteristics of the PM patients [3, 15, 31–33], and unfortunately could not be discarded in this study. As we demonstrated with 73 PM patients, combining cytogenetic and molecular analysis could contribute to a better understanding of the genetic aberrations and to obtain a better genotype-phenotype correlation in this group of patients [16].

## Conclusion

The search of the genetic origin of phenotypic manifestations in a heterogeneous group as PM patients, is an important challenge. PM patients with or without extracutaneous manifestations should be genetically characterized. As we showed in this study and considering the recommendations of other authors [3, 11, 27], the best strategy for the analysis of patients with pigmentary alterations is screening for chromosomal alterations in three different tissues in a large number of metaphases to discard low level mosaicism. As part of the global analysis, the application of molecular tools as microarrays or even NGS, is a necessary approach for the best genetic characterization of patients with PM. Moreover, the results obtained at the genetic level will contribute to get a better correlation with the clinical characteristics of the patient.

## Supplementary information

**Additional file 1.** “Cytogenetic alterations and molecular analysis detailed description”. The detailed descriptions of the abnormal karyotypes and molecular analysis in association with phenotype in 24 patients with chromosomal abnormalities.

## Data Availability

The data used and/or analysed to support the results of the current study are available from the corresponding author on reasonable request.

## Abbreviations

aCGH: array CGH
BAF: B allele frequencies
BL: Blaschko Lines
CA: Chromosomal Abnormalities
CNS: Central Nervous System
del: deletion
DFF: Dysmorphic Facial Features
DS: Hyperpigmentation
FISH: Fluorescence in situ Hybridization
ID: Intellectual Disability
IH: Hypopigmentation of Ito
LS: Hypopigmentation
LWNH: Linear and Whorled Hyperpigmentation
M-S: Musculoskeletal deformities
NGS: Next Generation Sequencing
PB: Peripheral Blood Lymphocytes
PM: Pigmentary Mosaicism
UPD: Uniparental Disomy
