# Supplementary material for: Genetic and clinical characterization of 73 Pigmentary Mosaicism patients: revealing the genetic basis of clinical manifestations
Source: Orphanet J Rare Dis. 2019 Nov 15;14:259. doi: 10.1186/s13023-019-1208-0 (PMC6858688; doi:10.1186/s13023-019-1208-0)
Supplement: Supplementary file 1 — Additional file 1. “Cytogenetic alterations and molecular analysis detailed description”. The detailed descriptions of the abnormal karyotypes and molecular analysis in association with phenotype in 24 patients with chromosomal abnormalities. [file 13023_2019_1208_MOESM1_ESM.pdf]

Additional file 1. Cytogenetic alterations and molecular analysis detailed description			
**Patient code**	**Clinical Manifestations**	**Karyotype**	**Molecular Analysis**
**PM2**	Broad BL pattern with disseminated hypo and hyperpigmentation<br>DD, ptosis, scoliosis, low weight and length and DFF	**PB:** mos 46, XY, r(7) (p22q36.3) [45]/45, XY,-7 [6]/46,XY, dup(7)(p22q36.3)[2]<br>**LS:** mos 45, XY, -7 [23]/46, XY r(7) (p22q36) [17]<br>**DS:** mos 45, XY, -7 [26]/46, XY r(7) (p22q36) [29]	**1.** arr 7p22.3(113,336–954,145)x1,7q36.1q36.3(151306863–158812247) x1, (Human Genome Build, hg18).<br>**2.** FISH analysis with ToTelVysion DNA probe mixture 7 (Abbot Molecular/Vysis)
**PM6**	Fine and whorled BL pattern with disseminated hypo and hyperpigmentation<br>DD, seizures and obesity	**PB:** mos 46, XY, del(18)(q21.3) [3]/46, XY [47]<br>**LS:** mos 46, XY, del(18)(q21.3) [11]/46, XY [39]<br>**DS:** mos 46, XY, del(18)(q21.3) [14]/46, XY [36]	NO
**PM8**	Fine and whorled BL pattern with disseminated hyperpigmentation, DD, hip dysplasia and low weight and length	**PB:** mos 69,XXY [2]/46,XY [49]<br>**LS:** 46, XY [50]<br>**DS:** mos 69,XXY [6]/46,XY [44]	NO
**PM9**	Fine and whorled BL pattern with disseminated hyperpigmentation, DD, hypotonia, DFF and RHH	**PB:** mos 46,XX, r(22) (p11.2q13.2)[15]/46,XX [40]<br>**LS:** mos 46, XX, r(22)(p11.2q13.2) [19]/46, XX [81]	NO
**PM13**	Fine and whorled BL pattern with disseminated hyperpigmentation, DD and seizures	**PB:** 46, XY, r(22)(p11.2q13.3) [100]<br>**LS:** mos 46, XY, r(22)(p11.2q13.3) [98]/46, XY, idic (22) [2]<br>**DS:** mos 46, XY, r(22)(p11.2q13.3) [96]/46, XY, idic (22) [4]	NO
**PM15**	Fine and whorled BL pattern with disseminated hyperpigmentation, severe DD, seizures, strabismus and digital alterations	**PB:** 46, XY [50]<br>**LS:** mos 47, XY, +i(12)(p10) [11]/46, XY [39]<br>**DS:** mos 47, XY, +i(12)(p10) [20]/46, XY [30]	NO
**PM25**	Fine and whorled BL pattern with disseminated hypo and hyperpigmentation, DD, DFF, scoliosis, hip dysplasia and low weight	**PB:** mos 47,XX,+mar[45]/47,XX, +14[10]/46,XX[45].<br>**LS:** mos 47,XX,+mar[7]/46,XX[8]<br>**DS:** mos 47,XX,+mar[12]/46,XX[14]	**1.** arr 14q11.1q11.2(18,127,052-19,927,052)x2~3, (UCSC, hg18)<br>**2.** FISH with DNA BACs probes for 14q11.2 spectrum green and 14q32.33 spectrum<br>**Final result:** PB: mos 47,XX,+del(14)(q11.2)[45]/47,XX,+14[10]/46,XX[45]
**PM27**	Fine and whorled BL pattern with disseminated hypo and hyperpigmentation, DFF and digital alterations	**PB:** 46,XX [50]<br>**LS:** 46,XX [50]<br>**DS:** mos 46, XX, t(1;8)(p?36;q?22)[11]/46,XX [139]	NO
**PM28**	Fine and whorled BL pattern with disseminated hyperpigmentation, DD, seizures, hypotonia, DFF and LHH	**PB:** mos 47,XY, +mar [4]/46, XY [47]<br>**LS:** 46, XY [25]<br>**DS:** 46, XY [50]	**1.** FISH with α-satellite 15 probe negative (Abbot Molecular/Vysis)

**PM30**	Fine and whorled BL pattern with disseminated hypo and hyperpigmentation, severe DD, seizures, hypotonia and DFF	**PB:** mos 47, XX,+mar [28]/45,X [6] / 46, XX [16]<br>**LS:** mos 45,X [35] / 47, XX,+mar [2]/ 46, XX [6]<br>**DS:** mos 45,X [20] / 47, XX,+mar [3]/ 46, XX [13]	**1.** arr Xp22.33q28(60,814-155,254,881)x1[~30%], Xp21.1p11.1(36,025,401-58,483,247)x3 [~55%] (Human Genome Build 37, hg19)<br>**2.** FISH with α-satellite X probe (AbbotMolecular/Vysis) and locus specific probe (p11.22-p11.23) (Agilent Tech, SureFISH) positive.<br>**Final result:** PB: mos 47, XX, +der(X)(p21.1p11.1) [28]/45,X [6] / 46, XX [16]
**PM39**	Fine and whorled BL pattern with disseminated hypo and hyperpigmentation, DD and low height	**PB:** mos 47,XY,+ mar [44]/ 46,XY [6]<br>**LS:** mos 47,XY,+ mar [2]/ 46,XY [38]<br>**DS:** mos 47,XY,+ mar [5]/ 46,XY [45]	**1.** FISH with α-satellite 14 probe (AbbotMolecular/Vysis) positive<br>**2.** arr (1-22)x2,(XY)x1, (UCSC, hg19) negative<br>**Final result:** marker probably of heterochromatic material
**PM41**	Fine and whorled BL pattern with disseminated hypopigmentation, severe DD, seizures, hypotonia, DFF scoliosis and low weight	**PB:** 46, X, t(X;22)(q11.2;q13.3) [50]<br>**LS:** 46, X, t(X;22)(q11.2;q13.3) [50]<br>**DS:** 46, X, t(X;22)(q11.2;q13.3) [50]	NO
**PM42**	Fine and whorled BL pattern with disseminated hypopigmentation, DD, seizures, DFF, strabismus, digital alterations and low weight and height,	**PB:** 46,XY,9qh- [50]<br>**LS:** mos 47, XY, 9qh-,+mar [19]/ 46,XY, 9qh- [33]<br>**DS:** mos 47, XY, 9qh-,+mar [9]/ 46,XY, 9qh- [42]	**1.** arr 9p12p11.2(43,504,857-44,259,464)x1, 9q21.11(69,092,561-69,972,419)x1, (UCSC, hg19)<br>**Final result:** marker probably of heterochromatic material
**PM46**	Fine and whorled BL pattern with localized hyperpigmentation, DD, iris heterochromia, DFF and digital alterations	**SP:** 46,XX, del(13)(q21.3q32.1) [50]<br>**LS:** 46,XX, del(13)(q21.3q32.1) [50]<br>**DS:** 46,XX, del(13)(q21.3q32.1) [50]	**1.**arr 13q21.31q32.1(63,958,459-96,671,274)x1, (UCSC, hg19)
**PM52**	Fine and whorled BL pattern with disseminated hypo and hyperpigmentation, DD, DFF and low height	**PB:** 46,XX,22pstk- [50]<br>**LS:** 46,XX,22pstk- [50]<br>**DS:** mos 47,XX, +12,22pstk-[44]/46,XX,22pstk- [6]	NO
**PM53**	Fine and whorled BL pattern with disseminated hyperpigmentation and DD	**PB:** 46,XX,del(4)(p16.1p15.3) [50]<br>**LS:** 46,XX,del(4)(p16.1p15.3) [50]<br>**DS:** 46,XX,del(4)(p16.1p15.3) [50]	**1.** arr 4p16.1p15.32 (10,047,353-17,614,303)x1, 8p22p21.3 (18,712, 712-19, 523, 339)x3, (UCSC, hg19)
**PM58**	Fine and whorled BL pattern with localized hyperpigmentation, severe DD, hypotonia, DFF and digital alterations	**PB:** 46,XY [50]<br>**LS:** mos 47, XY,+12[29]/ 46,XY [21]<br>**DS:** mos 47, XY,+12[32]/ 46,XY [19]	NO
**PM61**	Fine and whorled BL pattern with localized hyperpigmentation, DD, microphthalmia, DFF and	**PB:** 46, XX [50]<br>**LS:** 46, XX,-18,+mar [50]<br>**DS:** mos 46, XX,-18,+mar [45]/46, XX [5]	**1.** FISH FISH with α-satellite 18 probe (AbbotMolecular/Vysis) positive<br>**Final result:** 46,XX,-18, +mar.ish

	obesity		der(18)(D18Z1+)
**PM65**	Fine and whorled BL pattern with disseminated hyperpigmentation, DD and low weight	**PB:** mos 47,XY,+mar [48]/46,XY [2]<br>**LS:** mos 47,XY,+mar [46]/46,XY [4]<br>**DS:** mos 47,XY,+mar [27]/46,XY [23]	**1.** arr 15q11.1q11.2(20,394,272-22,759,378)x3, 15q11.2q13.2(22,759,378-30,509,325)x4 15q13.2q13.3(30,509,325-32,706,883)x3, (UCSC, hg19)
**PM67**	Fine and whorled BL pattern with localized hyperpigmentation, autistic features	**PB:** 47,XY, +mar [1] /46,XY [50]<br>**LS:** mos 46,XY, +mar1 [1]/47,XY,+mar2 [1]/45,XY,-12, +mar2 [1]/48,XY, +mar1, +mar2 [1]/46,XY [46]<br>**DS:** mos 44,XY,-1,-18,-20,+mar [1]/46,XY [14]	NO
**PM69**	Fine and whorled BL pattern, hyperpigmentation, DD, seizures, coloboma and low weight	**PB:** 46,XX [50]<br>**LS:** 46,XX [35]<br>**DS:** mos 47,XX,+?22 [3]/46,XX [47]	**1.** FISH with locus specific probe (22q11.2q11.2) (AbbotMolecular/Vysis) deletion negative
**PM71**	Fine and whorled BL pattern with localized hyperpigmentation, DD, LHH, cardiac alterations and low weight and length	**PB:** 46,XY [50]<br>**LS:** 46,XY, add(16)(p13.3) [25]<br>**DS:** mos 46,XY, add(16)(p13.3)[9]/46,XY [47]	NO
**PM72**	Broad BL pattern with disseminated hyperpigmentation, DD and low length	**PB:** 46,XY [50] 400-500B<br>**LS:** mos 47,XY,+12 [9]/46,XY [41]<br>**DS:** mos 47,XY,+12 [17]/46,XY [33]	NO

BL: Blaschko Lines; **DD:** Developmental Delay; **DFF:** Dysmorphic Facial Features; **PB:** Peripheral Blood; **LS:** Light Skin (Hypopigmented); **DS:** Dark Skin (Hyperpigmented); **RHH:** Right hemihypertrophy; **LHH:** Left hemihypertrophy.
NO: Molecular analysis not necessary or not realized yet.
